# Supplementary material for: Zfp296 knockout enhances chromatin accessibility and induces a unique state of pluripotency in embryonic stem cells
Source: Commun Biol. 2023 Jul 24;6:771. doi: 10.1038/s42003-023-05148-8 (PMC10366109; doi:10.1038/s42003-023-05148-8)

## Supplementary Information

### *Zfp296* knockout enhances chromatin accessibility and induces a unique state of pluripotency in embryonic stem cells

**Satsuki Miyazaki[1], Hiroyuki Yamano[2], Daisuke Motooka[2], Fumi Tashiro[3], Takumi Matsuura[1#], Tatsushi Miyazaki[1] & Jun-ichi Miyazaki[3*]**

[1]Division of Stem Cell Regulation Research, Center for Medical Research and Education, Osaka University Graduate School of Medicine, 2-2 Yamadaoka, Suita, Osaka 565-0871, Japan.
[2]Genome Information Research Center, Research Institute for Microbial Diseases, Osaka University, 3-1 Yamadaoka, Suita, Osaka 565-0871, Japan.
[3]The Institute of Scientific and Industrial Research (SANKEN), Osaka University, 8-1 Mihogaoka, Ibaraki, Osaka 567-0047, Japan.

*Correspondence should be addressed to J.M. (email: jimiyaza@nutri.med.osaka-u.ac.jp)
[#]Present address: Toray Industries, Inc., Tokyo, Japan.

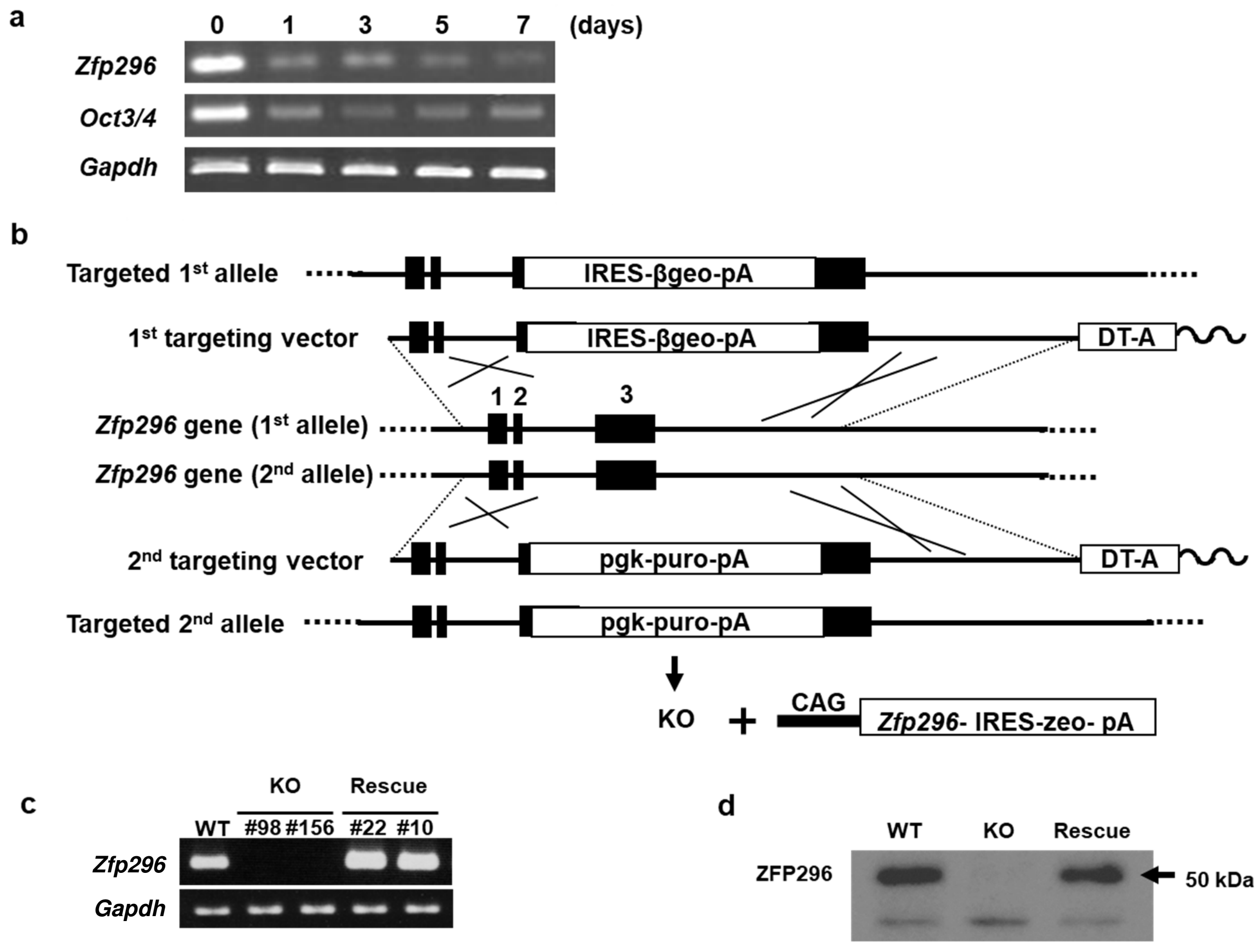


**Supplementary Fig. 1 Expression and targeted disruption of *Zfp296* in ESCs.** **a** Rapid decline of *Zfp296* expression after *Oct3/4* (also known as *Pou5f1*) suppression. ZHBTc4 ESCs lack both alleles of the *Pou5f1* gene and contain a *Pou5f1* transgene, whose expression is suppressed by tetracycline[20]. RNA was extracted from ZHBTc4 ESCs before and 1, 3, 5, and 7 days after the addition of tetracycline. **b** Targeted disruption of both alleles of the *Zfp296* gene. The *Zfp296* gene structure and the first and second targeting vectors are shown. **c** Analysis of *Zfp296* expression in WT, KO #98 and #156, and Rescue #22 and #10 ESCs by RT-PCR. **d** Western blot analysis of ZFP296 protein in WT, KO #98, and Rescue #22 ESCs using rabbit anti-ZFP296 antibody.

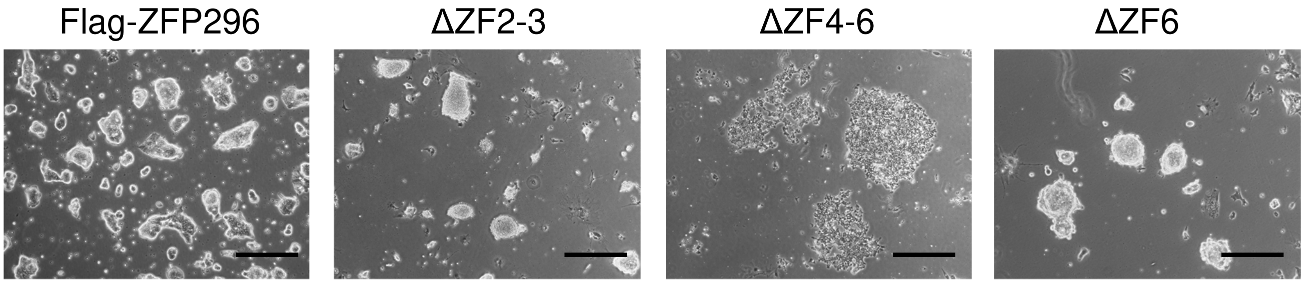


**Supplementary Fig. 2 Effects of ZFP296 deletion mutations on the colony morphology of ESCs.** KO #98 ESCs were transfected with pCAG-Flag-Zfp296, pCAG-Flag-Zfp296ΔZinc2-3, pCAG-Flag-Zfp296ΔZinc4-6, or pCAG-Flag-Zfp296ΔZinc6[15] and cultured in the presence of zeocin. Resulting colonies were pooled and replated. Their colony morphology was observed by phase-contrast microscopy. Scale bars, 300 μm.

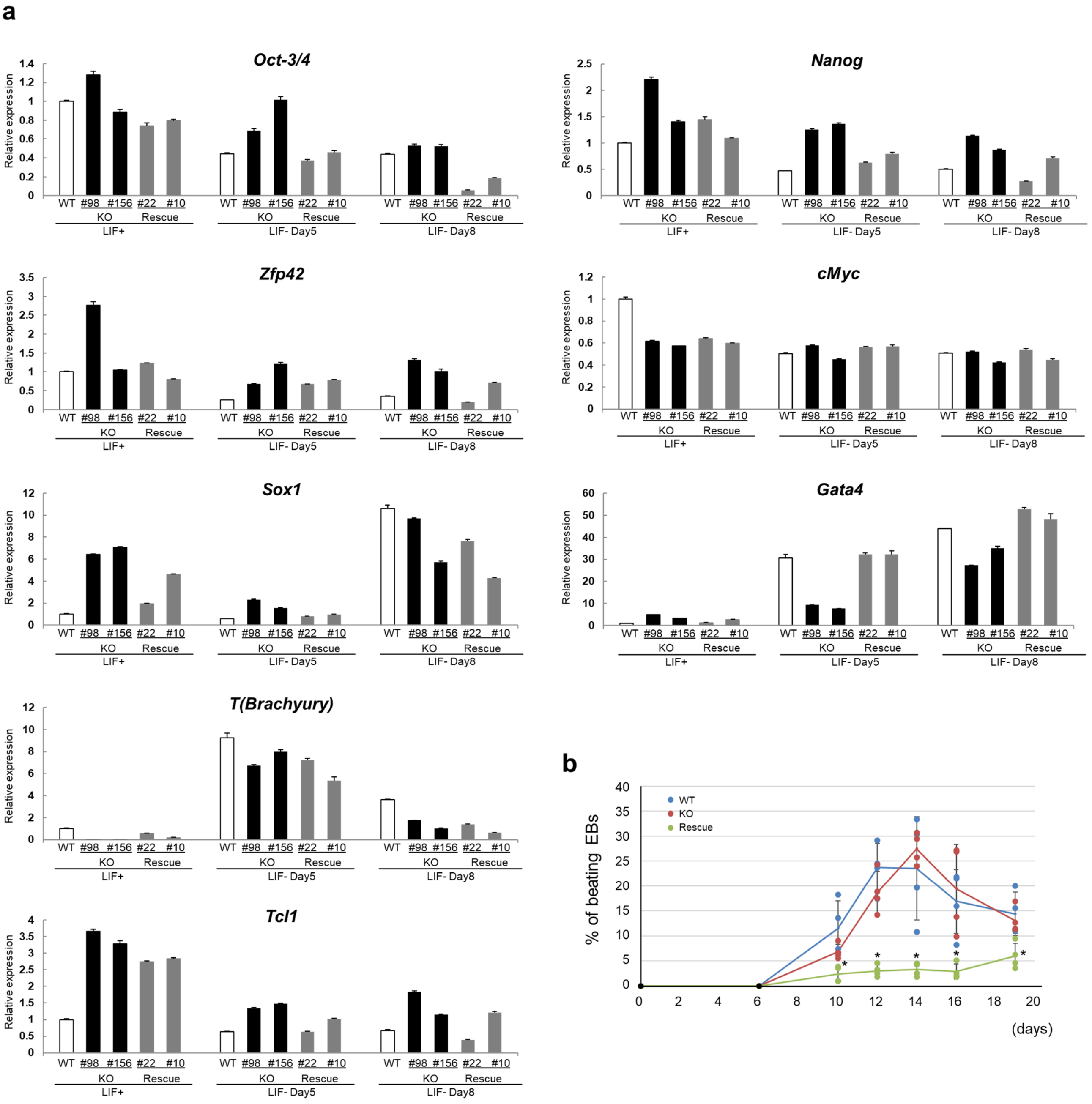


**Supplementary Fig. 3 Effects of *Zfp296* deficiency and overexpression on ESC differentiation *in vitro*. a** The expression of representative stem-cell and differentiation marker genes was examined by quantitative RT-PCR in WT, KO #98 and #156, and Rescue #22 and #10 ESCs grown in LIF(+) medium or in LIF(-) medium for 5 or 8 days. The expression level of each gene was measured relative to that in WT ESCs. Values are expressed as mean ± SD of three technical replicates. The expression of *Sox1* and *Gata4* was enhanced, but that of *T* (*Bra*) was reduced by *Zfp296* deficiency in LIF(+) culture. In LIF(-) culture, the expression of *Oct3/4* (*Pou5f1*) and *Nanog* was enhanced, but that of *Gata4* was reduced by *Zfp296* deficiency. **b** *In vitro* differentiation of WT, KO #98, and Rescue #22 ESCs into beating embryoid bodies (EBs). Cells were cultured in LIF(-) medium in bacterial dishes ($n = 4$). More than 100 EBs were formed in each dish. The percentage of beating EBs was measured at day 6 to day 19. Values are expressed as means ± SD. Difference from WT ESCs: $*P < 0.05$ by Tukey's test.

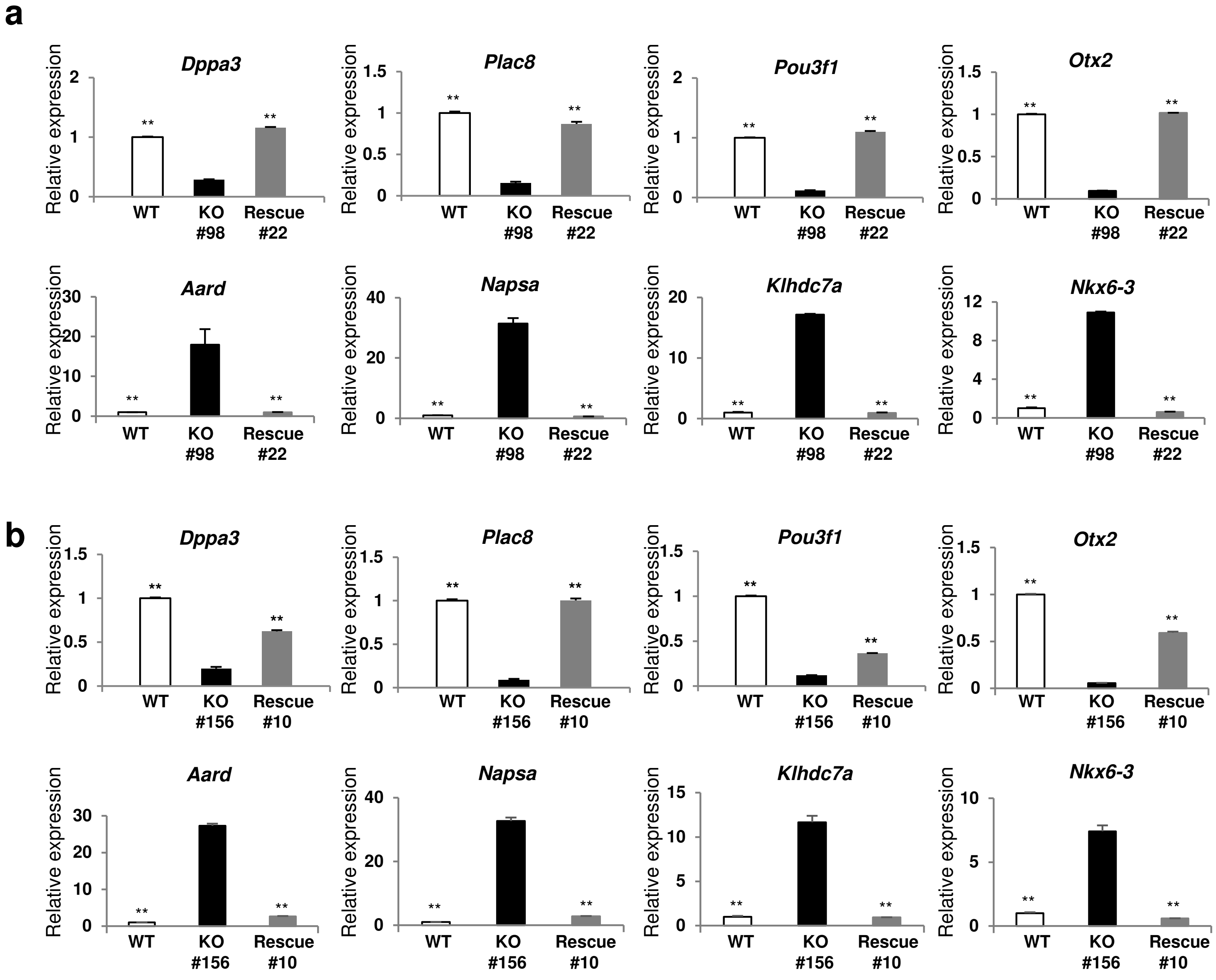


**Supplementary Fig. 4 Differentially expressed genes in the presence or absence of *Zfp296* expression.** **a, b** Quantitative RT-PCR analysis of the genes that were markedly downregulated (upper panels) or upregulated (lower panels) upon *Zfp296* KO in RNA-seq analysis. RNA was isolated from WT, KO #98, and Rescue #22 ESCs (**a**) or from WT, KO #156, and Rescue #10 ESCs (**b**). The expression level of each gene was normalized to that of *Actb* and shown as relative to that in WT ESCs. Values are presented as means ± SD of three technical replicates. Difference from KO cells: $**P < 0.01$ by Student's *t*-test.

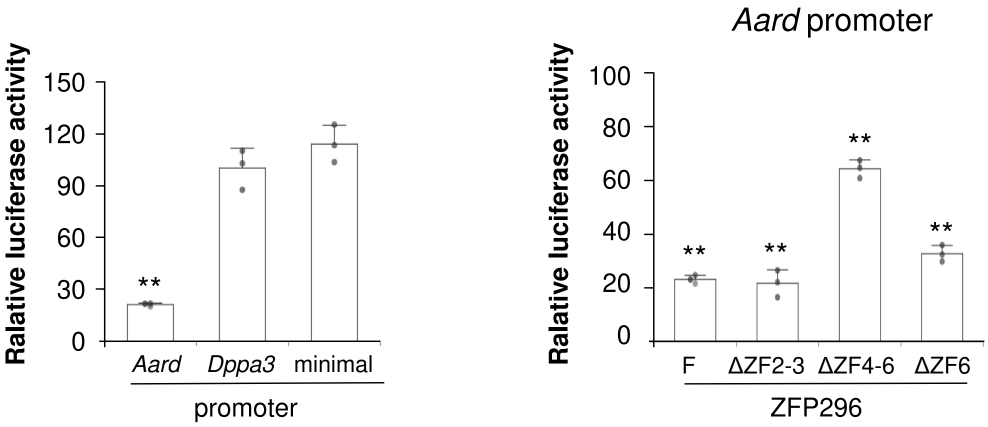


**Supplementary Fig. 5 Effects of ZFP296 deletion mutations on the repressor activity.** Luciferase assay was performed for the promoter regions of the mouse *Aard* and *Dppa3* genes. The left panel shows % luciferase activities directed by the *Aard*, *Dppa3*, or minimal promoter (pGL4.27) in the presence of ZFP296, relative to those in the absence of ZFP296. The right panel shows % luciferase activities directed by the *Aard* promoter in the presence of intact ZFP296 (F) or one of its deletion mutants (ΔZF2-3, ΔZF4-6, or ΔZF6), relative to those without ZFP296. **$P < 0.01$ vs. without ZFP296 by Student's *t*-test.

**Supplementary Table 1. Primers used for PCR.**

| Gene | Forward primer (5' to 3') | Reverse primer (5' to 3') | Reference |
|---|---|---|---|
| *Pou5f1* | CAGCCAGACCACCATCTGTC | GTCTCCGATTTGCATATCTCCTG | PrimerBank ID 7305399a3 |
| *Nanog* | TTGCTTACAAGGGTCTGCTACT | ACTGGTAGAAGAATCAGGGCT | PrimerBank ID 31338864a2 |
| *Zfp42* | CCAGCAGCTCCTGCACAC | GCCTATGACTCACTTCCAGGG | Miyazaki T et al. PLoS One 8(8):e71645, 2013 doi: 10.1371/journal.pone.0071645. |
| *c-Myc* | ATGCCCCTCAACGTGAACTTC | GTCGCAGATGAAATAGGGCTG | PrimerBank ID 293629266c1 |
| *Sox1* | CACAGTTCAGCCCTGAGTGA | CACAAACCACTTGCCAAAGA | Burgold T et al. PLoS One 3(8):e3034, 2008 doi: 10.1371/journal.pone.0003034. |
| *Pou5f1* | GGCGTTCTCTTTGGAAAGGTGTTC | CTCGAACCACATCCTTCTCT | Nichols J et al. Cell 95(3):379-91, 1998 doi: 10.1016/s0092-8674(00)81769-9. |
| *T* | GCTTCAAGGAGCTAACTAACGAG | | PrimerBank ID 6678203a1 |
| | | CGTCACGAAGTCCAGCAAGA | PrimerBank ID 118130357c1 |
| *Gata4* | TCAACCGGCCCCTCATTAAG | CACCCTCGGCATTACGACG | PrimerBank ID 6679953a3 |
| *Tcl1* | TTAATGTGGCAACTGTACCCC | TCCTCCACGTCTCTAAACTTGAT | PrimerBank ID 255918187c2 |
| *Dppa3* | GACCCAATGAAGGACCCTGAA | GCTTGACACCGGGGTTTAG | PrimerBank ID 21218416a1 |
| *Plac8* | GCTCAGGCACCAACAGTTATC | GCTGCCACTTGACATCCAAGA | PrimerBank ID 21105853a1 |
| *Pou3f1* | CAGTTCAAGCAACGACGCATC | CGAGAACACGTTACCGTAGAGG | PrimerBank ID 145279231c2 |
| *Otx2* | TATCTAAAGCAACCGCCTTACG | AAGTCCATACCCGAAGTGGTC | PrimerBank ID 21536266a1 |
| *Aard* | GAAGGGAGGAGGGGTGAG | GGCAAACTTTTAGTGCTTTGGT | Svingen T et al. Int J Dev Biol 51(3):255-8, 2007 doi: 10.1387/ijdb.062219ts. |
| *Nkx6-3* | GGGCGAGGCTGGCTTATTC | GGCTTGTTGTACTCATCGTCCT | PrimerBank ID 227908856c1 |
| *Napsa* | TAACCTCACAGGCCAGGACT | GCTTGGGGATATCCAAGGCTT | Khalifé M et al. PLoS One 6(8):e23253, 2011 doi: 10.1371/journal.pone.0023253. |
| *Klhdc7a* | CTGCGGCTTACAAACTCTACA | GAAGGCAAGTTGTCCCAAGG | PrimerBank ID 27734124a1 |
| *Actb* | AGTGTGACGTTGACATCCGTA | GCCAGAGCAGTAATCTCCTTCT | PrimerBank ID 6671509a3 |
| *Zfp296* | TCCAGTGTGGCAGACAGTAC | GGGCAGCACTGCTCACTGG | original |
| *Gapdh* | TGAAGGTCGGTGTGAACGGATTTGGC | CATGTAGGCCATGAGGTCCACCAC | Clontech Laboratories, Inc. |
| *Aard* promoter | ACCGGTACCTCTTACGCTACGGCTGACC | GGGCTCGAGGTTGCAAGTTGGAGCTTTT | original |
| *Dppa3* promoter | TTCTCGAGAAAGCATGAGCAGGTCTATCACAGC | TTGATATCCTTCACCTGAGCTACACCTTTAGGC | original |

**Supplementary Table 2. Antibodies used in the present study.**

| Antibodies | Source*; Cat# | Application** |
|---|---|---|
| Anti-Ty1-tag, mouse monoclonal | Diagenode; MAb-054-050 | ChIP-seq |
| Anti-H3K27me3, rabbit polyclonal | Upstate; 07-449 | WB |
| Anti-H3K9me1, mouse monoclonal | Active Motif; 39681 | WB |
| Anti-H3K9me2, rabbit monoclonal | Cell Signaling; 4658 | WB |
| Anti-H3K9me2, mouse monoclonal | Abcam; ab1220 | WB |
| Anti-H3K9me3, rabbit polyclonal | Abcam; ab8898 | WB |
| Anti-ZFP296, rabbit polyclonal | This study | WB |
| Anti-DPPA3, goat polyclonal | R&D Systems; AF2566 | WB, IF |
| Anti-Lamin B1, rabbit polyclonal | MBL; PM064 | WB |
| Anti-Histone H3, rabbit polyclonal | Abcam; ab1791 | WB |
| Anti-Flag M2, mouse monoclonal | Sigma-Aldrich; F3165 | IF |
| Anti-5mC, mouse monoclonal | Merck Millipore; 162 33 D3 | Dot blot |
| Anti-5hmC, rabbit polyclonal | Active Motif; 39769 | Dot blot |
| Anti-cleaved caspase-3 (Asp175), rabbit polyclonal | Cell Signaling; 9661 | IF |
| Anti-goat IgG-HRP, donkey polyclonal | Bethyl; A50-201P | WB |
| Anti-mouse IgG-HRP, goat polyclonal | MBL; 330 | WB |
| Anti-mouse IgG-HRP, horse polyclonal | Cell Signaling; 7076 | WB |
| Anti-mouse $IgG_1$-HRP, goat polyclonal | Bethyl; A90-105P | Dot blot |
| Anti-rabbit IgG-HRP, goat polyclonal | Cell Signaling; 7074 | WB |
| Anti-rabbit IgG-HRP, donkey polyclonal | Bethyl; A120-208P | Dot blot |
| Anti-goat IgG Alexa Fluor 488, donkey polyclonal | Molecular Probes; A11055 | IF |
| Anti-rabbit IgG Alexa Fluor 488, goat polyclonal | Molecular Probes; A11008 | IF |
| Anti-DDDDK-tag (Flag-tag), rabbit polyclonal | MBL; PM020 | WB |
| Anti-mouse $IgG_1$ Alexa Fluor 488, goat polyclonal | Molecular Probes; A21121 | IF |
| Anti-Myc-tag, rabbit polyclonal | MBL; 562 | WB |
| Anti-DYKDDDDK-tag (Flag-tag) mouse mAb-Magnetic Beads | FUJIFILM-Wako; 017-25151, 1E6 | IP |
| Anti-Myc-tag mouse mAb-Magnetic Beads | MBL; M047-11, PL14 | IP |

*R&D Systems, Minneapolis, MN
Bethyl, Montgomery, TX
**WB, western blotting; IP, immnunoprecipitation; IF, immunofluorescence

Supplementary Figure 6. Uncropped western blot images.

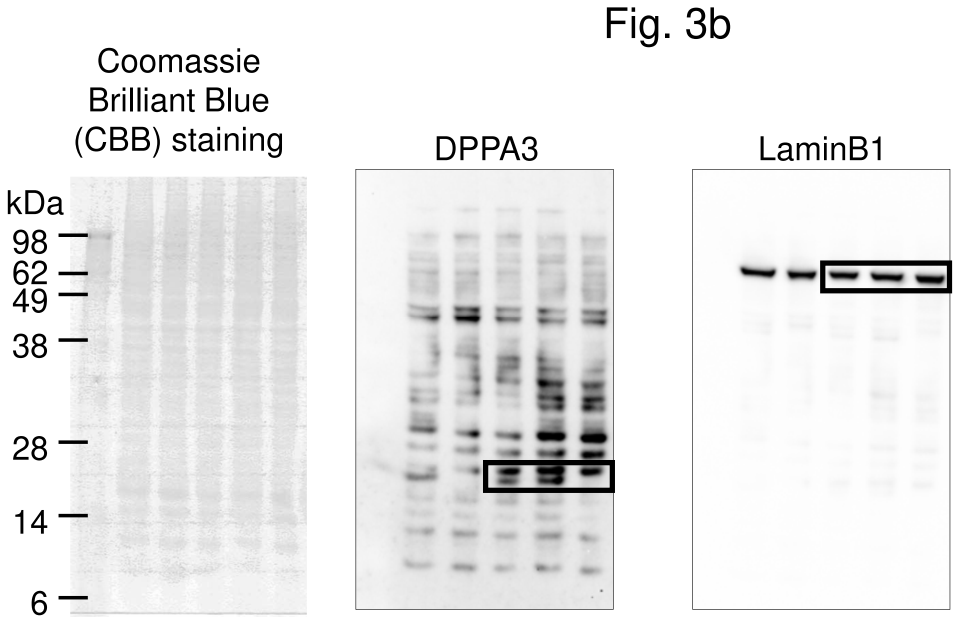

Fig. 7a

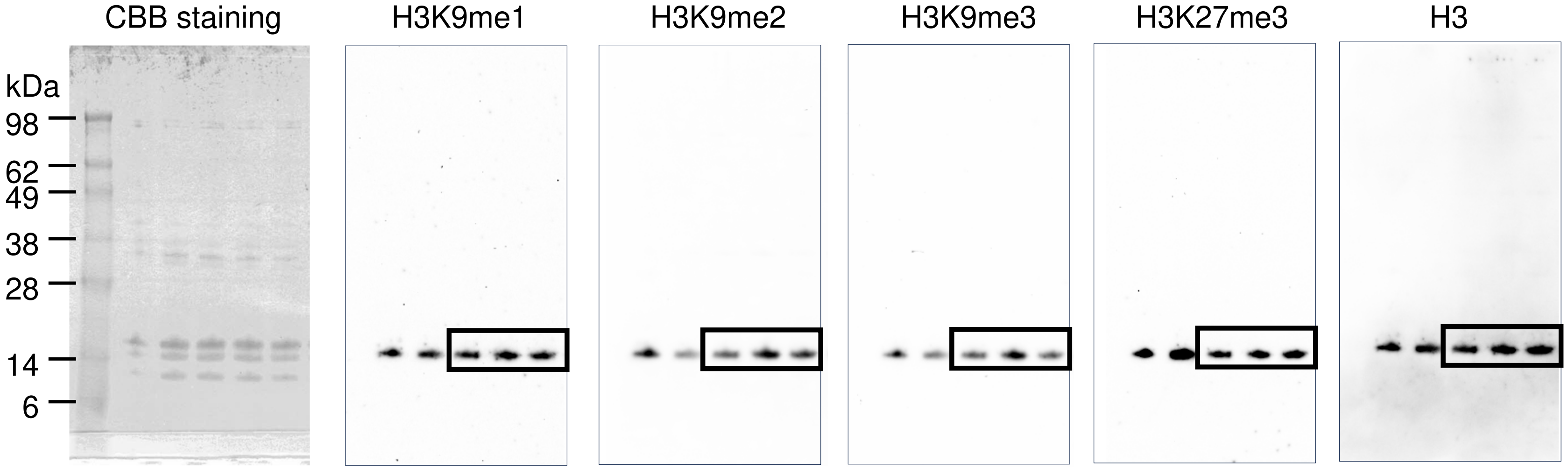
CBB staining
H3K9me1
H3K9me2
H3K9me3
H3K27me3
H3
kDa
98
62
49
38
28
14
6

Fig. 7b

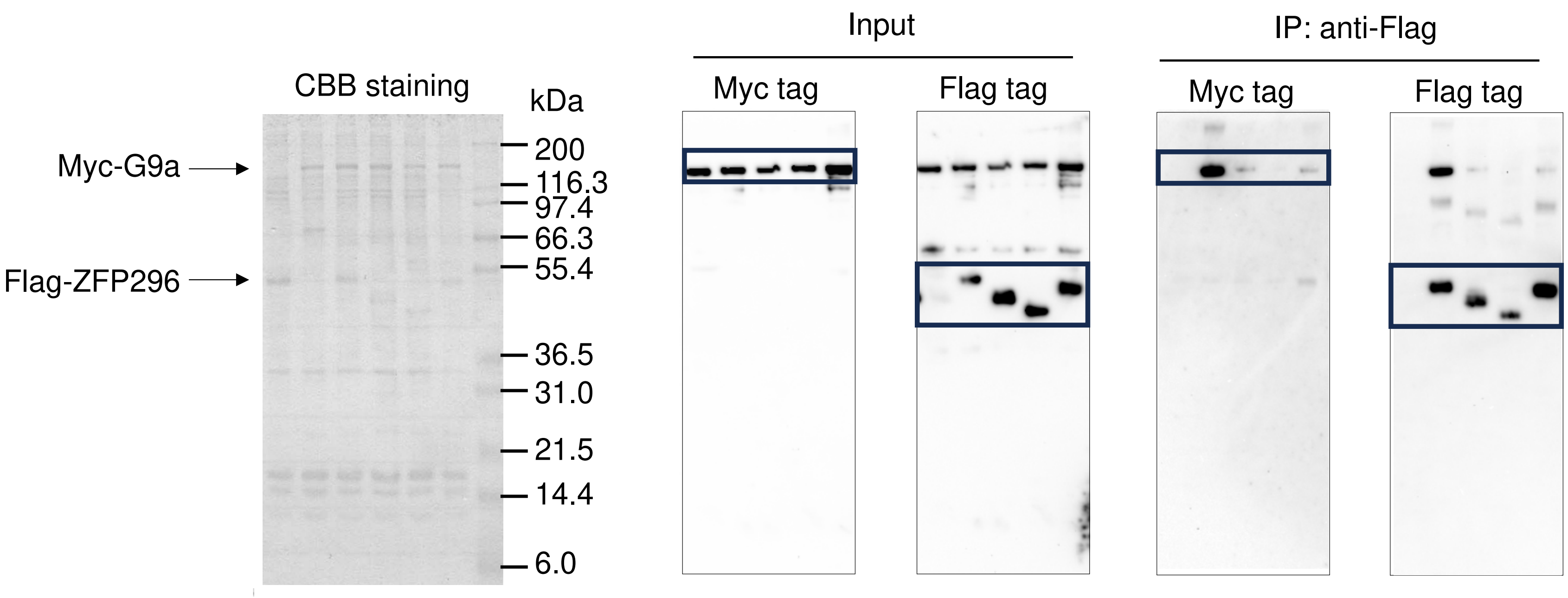

CBB staining
kDa
Myc-G9a
Flag-ZFP296
200
116.3
97.4
66.3
55.4
36.5
31.0
21.5
14.4
6.0
Input
IP: anti-Flag
Myc tag
Flag tag
Myc tag
Flag tag

Fig. 7c

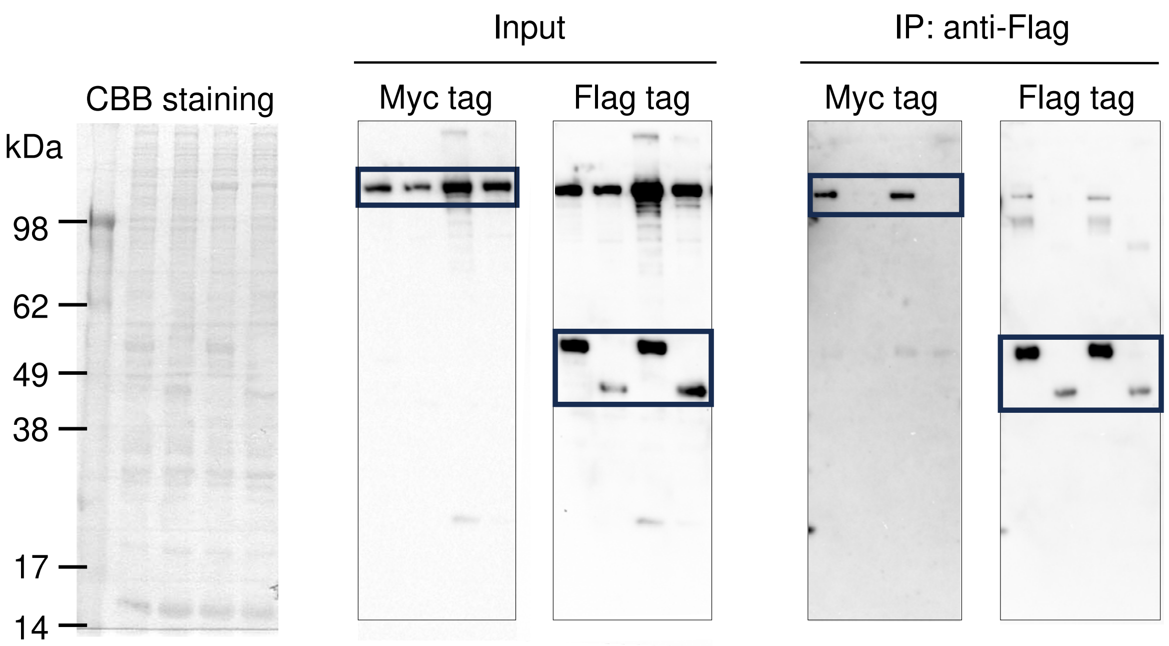
Input
IP: anti-Flag
CBB staining
Myc tag
Flag tag
Myc tag
Flag tag
kDa
98
62
49
38
17
14

Fig. 7d

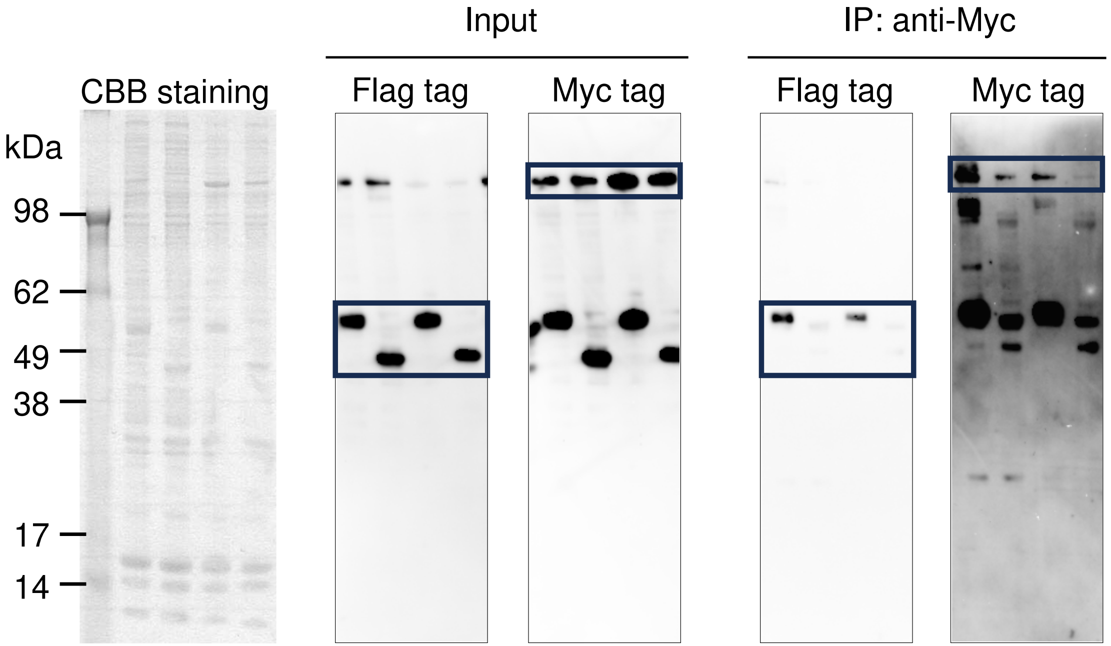
Input
IP: anti-Myc
CBB staining
Flag tag
Myc tag
Flag tag
Myc tag
kDa
98
62
49
38
17
14

Supplementary Fig. 1d

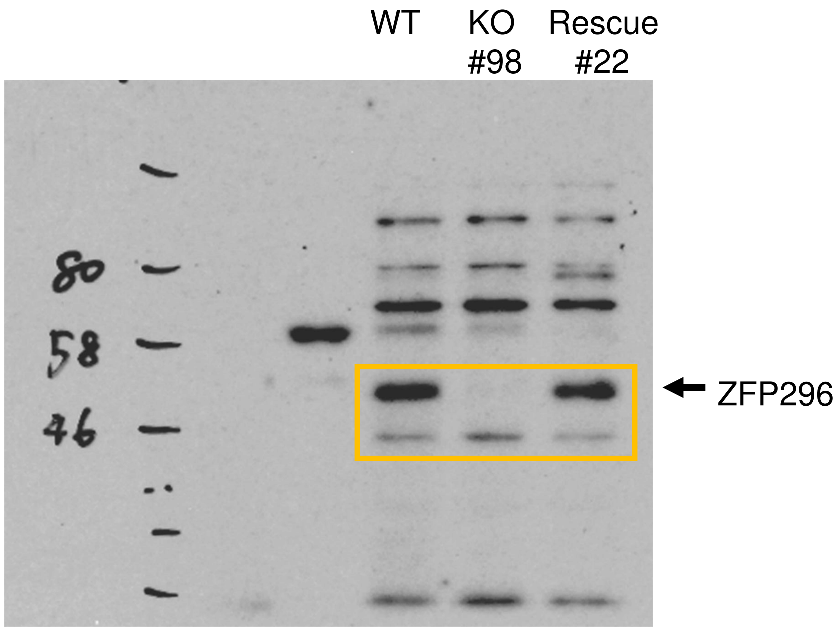

Supplement: Supplementary file 2 — Supplementary Information [file 42003_2023_5148_MOESM2_ESM.pdf]
