# Supplementary material for: Zfp296 knockout enhances chromatin accessibility and induces a unique state of pluripotency in embryonic stem cells
Source: Commun Biol. 2023 Jul 24;6:771. doi: 10.1038/s42003-023-05148-8 (PMC10366109; doi:10.1038/s42003-023-05148-8)
Supplement: Supplementary file 5 — Reporting Summary [file 42003_2023_5148_MOESM5_ESM.pdf]

nature portfolio

Corresponding author(s): Jun-ichi Miyazaki

Last updated by author(s): Jul 11, 2023

Reporting Summary

Nature Portfolio wishes to improve the reproducibility of the work that we publish. This form provides structure for consistency and transparency in reporting. For further information on Nature Portfolio policies, see our Editorial Policies and the Editorial Policy Checklist.

Statistics

For all statistical analyses, confirm that the following items are present in the figure legend, table legend, main text, or Methods section.

n/a	Confirmed	
☐	☒	The exact sample size (*n*) for each experimental group/condition, given as a discrete number and unit of measurement
☐	☒	A statement on whether measurements were taken from distinct samples or whether the same sample was measured repeatedly
☐	☒	The statistical test(s) used AND whether they are one- or two-sided<br>*Only common tests should be described solely by name; describe more complex techniques in the Methods section.*
☒	☐	A description of all covariates tested
☐	☒	A description of any assumptions or corrections, such as tests of normality and adjustment for multiple comparisons
☐	☒	A full description of the statistical parameters including central tendency (e.g. means) or other basic estimates (e.g. regression coefficient) AND variation (e.g. standard deviation) or associated estimates of uncertainty (e.g. confidence intervals)
☐	☒	For null hypothesis testing, the test statistic (e.g. *F*, *t*, *r*) with confidence intervals, effect sizes, degrees of freedom and *P* value noted<br>*Give P values as exact values whenever suitable.*
☒	☐	For Bayesian analysis, information on the choice of priors and Markov chain Monte Carlo settings
☒	☐	For hierarchical and complex designs, identification of the appropriate level for tests and full reporting of outcomes
☒	☐	Estimates of effect sizes (e.g. Cohen's *d*, Pearson's *r*), indicating how they were calculated

Our web collection on statistics for biologists contains articles on many of the points above.

Software and code

Policy information about availability of computer code

Data collection	TopHat ver. 2.0.13, Subread featureCounts v2.0.3, Stampy with default parameters, MACS2 software, Cutadapt v3.2, Bowtie2 v2.3.5.1, Picard tools v1.128, deepTools v3.4.3 alignmentSieve
Data analysis	edgeR v3.36.0 in R v4.1.2, Cufflinks, Cis-regulatory Element Annotation System (CEAS), MEME, DREME, Bedtools v2.25.0, HOMER, Subread featureCounts v2.0.3

For manuscripts utilizing custom algorithms or software that are central to the research but not yet described in published literature, software must be made available to editors and reviewers. We strongly encourage code deposition in a community repository (e.g. GitHub). See the Nature Portfolio guidelines for submitting code & software for further information.

Data

Policy information about availability of data

All manuscripts must include a data availability statement. This statement should provide the following information, where applicable:

- Accession codes, unique identifiers, or web links for publicly available datasets
- A description of any restrictions on data availability
- For clinical datasets or third party data, please ensure that the statement adheres to our policy

The data supporting the findings of this study are available from the corresponding author upon reasonable request.

March 2021

Sequencing data of RNA-seq, ChIP-seq, and ATAC-seq generated in this study have been deposited in the NCBI GEO repository under the accession number GSE231412.

Human research participants

Policy information about studies involving human research participants and Sex and Gender in Research.

Reporting on sex and gender	N/A
Population characteristics	N/A
Recruitment	N/A
Ethics oversight	N/A

Note that full information on the approval of the study protocol must also be provided in the manuscript.

Field-specific reporting

Please select the one below that is the best fit for your research. If you are not sure, read the appropriate sections before making your selection.

☒ Life sciences ☐ Behavioural & social sciences ☐ Ecological, evolutionary & environmental sciences

For a reference copy of the document with all sections, see nature.com/documents/nr-reporting-summary-flat.pdf

Life sciences study design

All studies must disclose on these points even when the disclosure is negative.

Sample size	Sample sizes were not predetermined by statistical methods. We employed standard sample sizes based on previous experiments and literature including similar experiments. These sample sizes were thought enough to detect the significant differences among the groups.
Data exclusions	In gene expression analysis based on RNA-seq, we excluded genes whose read counts were very low and noncoding genes as described in the figure legend.
Replication	The results presented were reproduced in multiple experiments.
Randomization	N/A
Blinding	N/A

Reporting for specific materials, systems and methods

We require information from authors about some types of materials, experimental systems and methods used in many studies. Here, indicate whether each material, system or method listed is relevant to your study. If you are not sure if a list item applies to your research, read the appropriate section before selecting a response.

Materials & experimental systems

n/a	Involved in the study	
☐	☒	Antibodies
☐	☒	Eukaryotic cell lines
☒	☐	Palaeontology and archaeology
☐	☒	Animals and other organisms
☒	☐	Clinical data
☒	☐	Dual use research of concern

Methods

n/a	Involved in the study	
☐	☒	ChIP-seq
☒	☐	Flow cytometry
☒	☐	MRI-based neuroimaging

Antibodies

Antibodies used	Antibodies used in this study are listed in Supplementary information, which provides the supplier name and catalog number for each antibody.

Validation	Supplementary information also provides the species, monoclonal or polyclonal, and application for each antibody used.

Eukaryotic cell lines

Policy information about cell lines and Sex and Gender in Research

Cell line source(s)	Murine embryonic stem (ES) cell line EB3 derived from E14tg2a and human embryonic kidney cell line 293T.
Authentication	The EB3 cell line is a germ line-competent ES cell line obtained from Professor Hitoshi Niwa at Kumamoto University (Niwa et al, Mol. Cell. Biol. 22, 1526–1536, 2002).<br>The 293T cell line was purchased from Clontech (Cat# 632180).
Mycoplasma contamination	EB3 and 293T cells were confirmed to be negative for mycoplasma contamination.
Commonly misidentified lines (See ICLAC register)	N/A

Animals and other research organisms

Policy information about studies involving animals; ARRIVE guidelines recommended for reporting animal research, and Sex and Gender in Research

Laboratory animals	Nude mice, (C57BL/6J x 129/Ola) F1 mice, C57BL/6J mice, and ICR mice at the age of ~ 8 weeks.
Wild animals	N/A
Reporting on sex	N/A
Field-collected samples	N/A
Ethics oversight	The Institutional Animal Care and Use Committee of Osaka University

Note that full information on the approval of the study protocol must also be provided in the manuscript.

ChIP-seq

Data deposition

☒ Confirm that both raw and final processed data have been deposited in a public database such as GEO.

☒ Confirm that you have deposited or provided access to graph files (e.g. BED files) for the called peaks.

Data access links<br>*May remain private before publication.*	Sequencing data of RNA-seq, ChIP-seq, and ATAC-seq generated in this study have been deposited in the NCBI GEO repository under the accession number GSE231412.
Files in database submission	GSE231392, GSE231393, GSE231410, GSE231411
Genome browser session (e.g. UCSC)	https://www.ncbi.nlm.nih.gov/geo/query/acc.cgi?acc=GSE231412

Methodology

Replicates	Single analysis was performed considering the sequencing depth and the resulting accuracy of next-generation sequencing.
Sequencing depth	101-base single reads, total 22 million reads
Antibodies	anti-Ty1-tag monoclonal antibody (Diagenode, Denville, NJ) , MAb-054-050
Peak calling parameters	Sequence reads were mapped to the mm10 mouse genome using Stampy with default parameters. Peak calling was performed using MACS2 software with default parameters to call areas of enrichment relative to the genome background.
Data quality	Peaks with q-value < 0.1 were identified. The number of binding peaks was 3273.
Software	Softwares used: Stampy with default parameters, MACS2, Cis-regulatory Element Annotation System (CEAS), MEME, and DREME softwares.
